# Novel *Theileria* sp. as an Etiology of Cutaneous Theileriosis among the Vulnerable Arabian Oryx

**DOI:** 10.3390/pathogens13060485

**Published:** 2024-06-07

**Authors:** Sonia Boughattas, Mutassim A. Salih, Andrea Dogliero, Nahla O. Eltai

**Affiliations:** 1 Biomedical Research Center, Qatar University, Doha P.O. Box 2713, Qatar; sbgh@mail.com (S.B.); mutassim.salih@qu.edu.qa (M.A.S.); 2 Department of Natural Reserves, Ministry of Environment and Climate Change, Doha P.O. Box 7634, Qatar; andrea.dogliero@gmail.com

**Keywords:** *Theileria* sp., wildlife, Arabian oryx, microscopy, 18S sequencing

## Abstract

The Arabian Peninsula’s endemic ungulate, *Oryx leucoryx*, was on the verge of extinction at the end of the 1970s. Despite the different reintroduction programs, the International Union for Conservation of Nature is still classifying it as Vulnerable. Among other factors, their vulnerability lies in their susceptibility to specific etiological agents that affect livestock, necessitating health monitoring and strict preventive/biosecurity measures. Within this frame, the current work investigated the determination of the etiological agent potentially involved with cutaneous lesions observed in eight males of Arabian oryx within one of the several national governance conservation programs. Microscopic examination from one animal specimen suggested theileriosis association, which was confirmed by molecular tools using 18S gene sequencing and the report of a novel *Theileria* sp. not clustering with previously reported antelope sequences. This finding prompts further explorations into the disease dynamics within the Arabian oryx population, especially with the scarcity of data in Qatar about tick-borne pathogens and their transmission.

## 1. Introduction

The white or Arabian oryx (*Oryx leucoryx*) is an endemic ungulate of the Arabian Peninsula, hence its name. It is also the largest adapted antelope and inhabits the harsh desertic environment [1]. Traditionally, the animals were hunted for their meat and hides; however, the hunting pressure, in addition to environmental degradation due to the massive construction and development in the region, played a significant role in the extinction of this species in the wild by 1972 [2]. Since then, conservation efforts have been deployed to rescue the Arabian oryx from extermination by establishing successful captive breeding programs within the Arabian Peninsula [3]. The number of Arabian oryx is estimated globally to be 1,100 in the wild and even more in captivity, within private collections, government reserves, and international zoos [4]. Despite such efforts, according to the International Union for Conservation of Nature (IUCN), the Arabian oryx is classified within the Red List of Threatened Species as Vulnerable under criterion D1 [5].

Health monitoring is consequently crucial to secure the species’ conservation, especially when there is evidence of the Arabian oryx’s susceptibility to specific etiological agents known to affect livestock [6], presenting a risk of infection to the relocated animals either with previously present agents or by introducing novel pathogens into naïve regions [7]. Previous epidemiological studies that targeted the causes of different diseases by screening the oryx were limited to bacteria [8,9], including *Mycobacterium* [10,11] and *Mycoplasma* [12]; mites [5,13]; parasites (both helminths [14] and protozoa [15,16,17]); prions [18,19]; and viruses [2,20,21,22] as the causative agents investigated. No data are available on other zoonotic or tick-borne pathogens that may affect the Arabian oryx’s health. To address these gaps, using a molecular epidemiology approach, we investigate in the present study the relevant causative agents of cutaneous alterations observed within Qatari *Oryx leucoryx* cases.

## 2. Materials and Methods

### 2.1. Sampling

**Figure 1:**
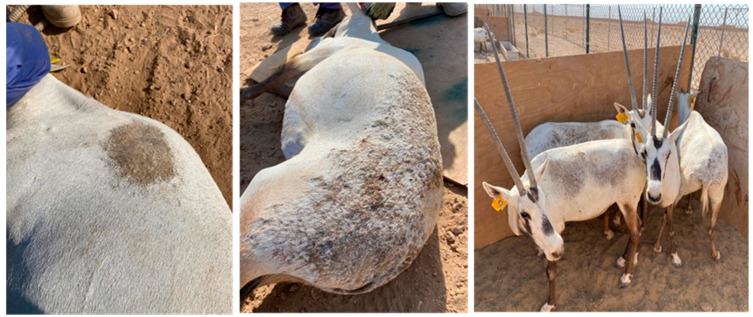
Wild captive males of Arabian oryx presenting skin lesions.

Blood and skin tissue samples were collected in January 2021 from a government breeding conservation center in Qatar by a veterinarian doctor, complying strictly with the National Institutional Animal Care and Use Committee’s animal welfare guidelines. Specimens were sampled from eight male Arabian oryx, aged two years old, demonstrating skin lesions associated with respiratory and enteric signs (Figure 1). Approximately 5 mL of blood was collected from the jugular vein in Ethylenediamine Tetraacetic Acid (EDTA) tubes. From each subject, an incisional biopsy of 0.5 cm^3^ was surgically taken with a scalpel blade by ring infiltration of one local anesthetic (Lidocaine 1%), always taking care not to include a portion of healthy skin in the sample. Each biopsy was appropriately deposited in a sterile serum tube. After collection, the samples were stored in cool boxes (4–8 °C) and directly transported to the microbiology laboratory at the Biomedical Research Center (BRC), Qatar University, for consequent analysis. Qatar University’s Institutional Biosafety Committee (IBC) granted permission to conduct the study under approval number QU (QUIBC2019/060-REN1).

### 2.2. Diagnosis

#### 2.2.1. Microscopy

Briefly, thin and thick blood films were prepared for microscopical examination. The thin blood smear was generated by placing a drop of EDTA anticoagulated blood on one-third of a glass slide and spreading it at a 45-degree angle with another slide. All thin blood smears were then air-dried for 5 min, fixed with methanol for 2 min, stained with 10% Giemsa (Anamol, Palghar, India) for 10 min, washed with water, and dried. Subsequently, the smears were examined under a microscope at ×100 magnification (oil immersion lens) for parasite detection. In contrast, the thick blood film was prepared by placing a drop of blood in the empty third of the glass slide and spreading circularly. The smears were not fixed with methanol or heat, and they hence consisted of a thick layer of lysed red blood cells, thus allowing more efficient detection of parasites with increased sensitivity.

#### 2.2.2. Molecular Analysis

From the collected blood specimens and skin biopsies, DNA extraction was performed using a DNeasy Blood & Tissue Kit (Qiagen, Hilden, Germany) followed by protists screening via PCR reaction targeting the common eukaryotic V8 small subunit ribosomal DNA with the primer sets 1427F TCTGTGATGCCCTTAGATGTTCTGGG and 1616R GCGGTGTGTACAAAGGGCAGGG [23]. PCR amplification was performed using 2X HotStart Taq plus master mix (Qiagen) and 0.5 mM of each primer in a total reactional volume of 25 μL according to the cycling conditions of initial denaturation for 5 min at 95 °C followed by 25 cycles of 94 °C for 30 s, 52 °C for 60 s, and 68 °C for 90 s, with a final extension step for 10 min.

Further molecular investigations employed the primer sets ITS1F TGACATTTAATAACAATCAACCCTT and ITS1R GGTTTGTATTAACCAATCCGTGA [24] as well as the 18S set Ts1(F) CACTCCAACAGTCGCCCACAGAC; Ts2(R) CAGCGCTGAGGACGG CAAGTG [25]. Negative and no-template controls were included in each amplification run. The end products of the different amplifications were purified and sent for direct bi-directional Sanger sequencing to Macrogen© (Seoul, Republic of Korea). Visualization, cleaning, and editing of the sequences was achieved by BioEdit v7.2.5 software. The consensus sequences were then subjected to BLAST analysis to assess the homologous sequences with the already known ones deposited in the NCBI database. Multiple alignment with observed similar sequences was performed using MAFFT v7 software and the obtained aligned sequences were run for phylogenetic analysis with the *Cardiosporidium cionae* sequence (Accession number: EU052685.1) used as an outgroup by MEGA X following the Maximum-Likelihood ML approach with 1000 bootstraps. The nucleotide sequences from the current study have been deposited into the GenBank database under accession numbers OR921373 and OR921374.

## 3. Results

**Figure 2:**
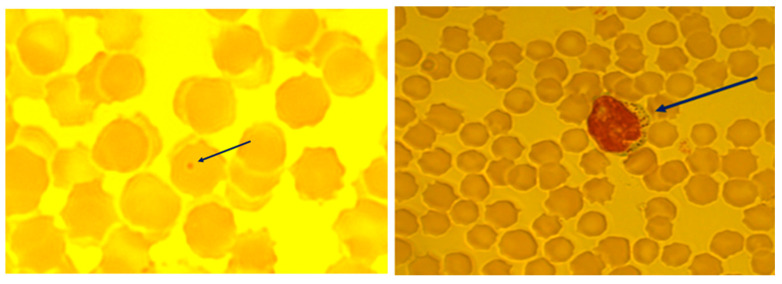
(**Left**) Thin blood smear showing schizont (Koch’s blue bodies) of *Theilaria* sp. in Arabian oryx’s white blood cells (lymphocyte). (**Right**) Infected erythrocyte showing *Theileria* sp. piroplasm.

Under a light microscope with an oil immersion lens (×100), a Giemsa-stained blood smear of Arabian oryx blood suggested the presence of typical *Theilaria* sp. schizont in the lymphocytes and piroplasm and merozoites in the erythrocytes (Figure 2). However, such an observation was recorded in 12.5% of the investigated subpopulation within only one of eight specimens. This approach was a gold-standard technique before the development of molecular tools [26].

The initial amplification reaction was successful with the observation of the ≈180 bp band after agarose electrophoresis for all the analyzed specimens. The corresponding sequences generated were unambiguous, and when compared among the different blood and skin specimens, an identical sequence was observed. The BLAST of the consensus sequence (Accession number: OR921373) revealed an exact match with 100% for Query Cover and Percent Identity for both *Theileria velifera* (Accession number: OQ818179) and *Besnoitia besnoiti* (Accession number: FN435990.1) isolates. No amplifications were observed using the specific ITS primers for *Besnoitia* sp. identification, which discarded the potential involvement of the *Besnoitia* parasite in our cutaneous cases, whereas successful amplification and sequencing were recoded with the 18S set of primers targeting *Theileria* species (Accession number: OR921374).

**Figure 3:**
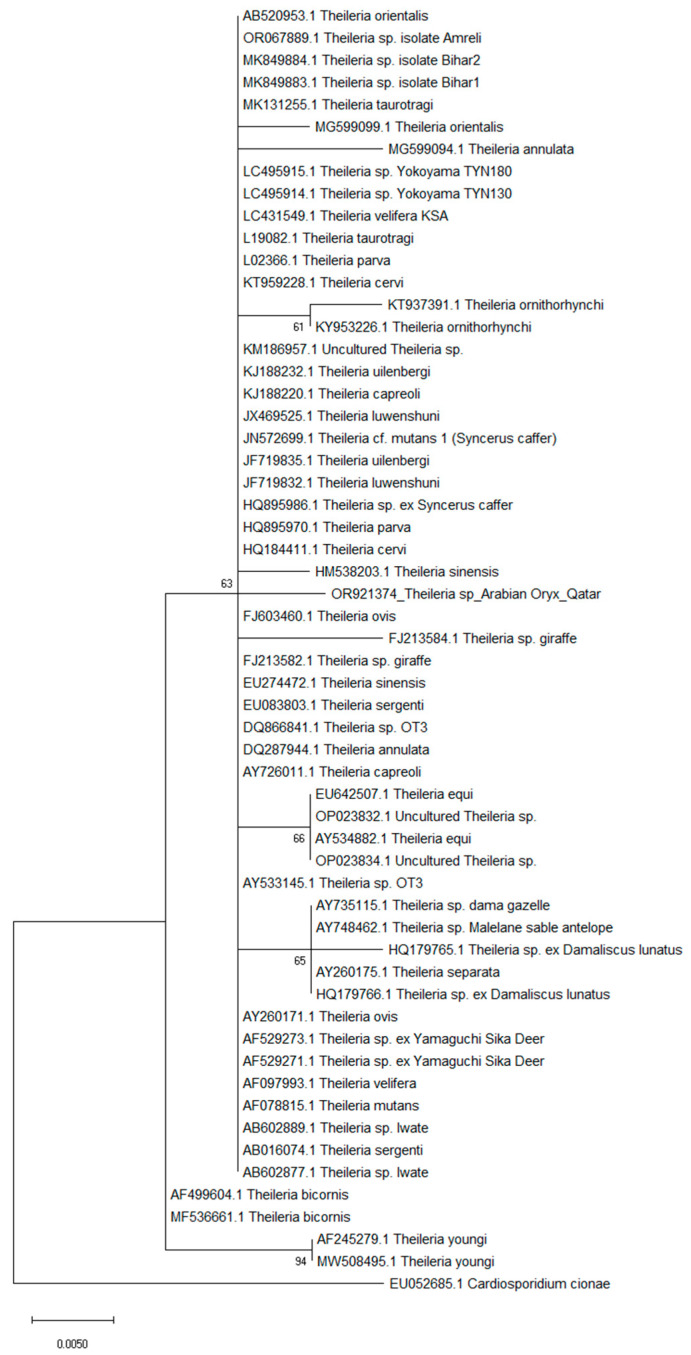
The Maximum Likelihood (K2 substitution model) phylogenetic tree based on the analysis of the partial 18 SSU-rDNA gene of *Theileria* isolates. Numbers next to the nodes represent posterior probabilities.

Since the 18S RNA is a highly conserved gene which enables subsequent genetic diversity analysis [27], we used the generated sequences to explore our isolate’s phylogenetic position within 64 *Theileria* sp. retrieved from the GenBank database. Our oryx isolate did not cluster with previously reported sequences from different antelope species, such as the African (Accession numbers: HQ179766.1; HQ179765.1) and Sable (Accession number: AY748462.1) antelopes, but bunched distinctly on a separate phylogenetic tree branch (63%). It is worth noting that our isolate sequence did not bundle with the common *Theileria* species reported previously within herbivore animals identified from the MENA region (Accession numbers: AY735115; LC431549) but were surrounded by *Theileria* species identified from China (HM538203.1; FJ603460.1) (Figure 3).

## 4. Discussion

So far, around 20 species of the apicomplexan protozoa *Theileria* sp. have been reported worldwide as etiological agents of theileriosis that vary widely in virulence, ranging from severe diseases like East Coast Fever, resulting in high levels of mortality, to completely benign and even asymptomatic forms [28]. Cutaneous cases with hemorrhagic and/or necrotic lesions are, however, scattered and reported so far only within bovine theileriosis [29,30,31], which supports our observations since antelopes are classified as Bovidae. Nevertheless, theileriosis mainly affects small ruminants, Cervidae, Capridae, and New World Camelidae, as well as wild mammals [32] and the Bovidae, Equidae, and other domestic hosts. Among the causative species, *T. annulata*, *T. lestoquardi*, *T. luwenshuni*, *T. parva*, and *T. uilenbergi* are considered highly pathogenic. In contrast, the rest of the species are considered scarcely or not pathogenic, even if benign [28]. Most *Theileria* sp. advocate the host specificity concept. However, some species show a wider distribution, for instance, as reported elsewhere for *Theileria* sp. (sable) identified within African buffalo, cattle dogs, and antelopes [33].

The parasite species are transmitted by hard tick (ixodid) vectors, leading to a Tick-borne Disease (TBD) that has become a growing focus of attention recently, as a significant life-threatening veterinary concern to wildlife and domesticated animals, as well as clinically affecting general human health [34,35]. Each species seems restricted to a specified geographical zone, with particular genera of ticks reported to act as vectors for particular *Theileria* sp., such as *Amblyomma* as a vector for *T. mutans* and *T. velifera*; *Haemaphysalis* as a vector for *T. orientalis*, *T. uilenbergi*, and *T. luwenshuni*; *Hyalomma* as vector for *T. annulata*, *T. lestoquardi*, and *T. separata*; and *Rhipicephalus* as a vector for *T. parva*, *T. taurotragi*, *T. ovis*, and *T. lestoquardi* [36]. The MENA region has suitable climates as well as favorable conditions for the expansion of ticks and associated TBDs [37,38] due to widespread livestock ranching, the import of animals from other geographical territories, the wildlife population’s abundance supporting ticks’ lifecycles, and climate change conditions [39]. However, studies on TBDs within the State of Qatar are still scarce, with only reports of *Hyalomma dromedarii* [40], *Hyalomma impeltatum* [40], and more recently *Hyalomma aegyptium* vectors [41].

## 5. Conclusions

Since wild animals are well-established reservoir hosts of TBD, their migration, trading, and transportation enhance pathogens’ spread. Based on the current work’s results in identifying a novel pathogenic *Theileria* sp. within the vulnerable Arabian oryx and the scarcity of data about its etiological agent, further investigations targeting the vector’s identification and transmission are crucial for the health management of vulnerable herds and their reintroduction programs.

## Data Availability

Supporting data were submitted to GenBank under Accession numbers OR921373 and OR921374.
